# Melatonin exerts anti-oral cancer effect via suppressing LSD1 in patient-derived tumor xenograft models

**DOI:** 10.18632/oncotarget.16808

**Published:** 2017-04-04

**Authors:** Cheng-Yu Yang, Chih-Kung Lin, Chang-Huei Tsao, Cheng-Chih Hsieh, Gu-Jiun Lin, Kuo-Hsing Ma, Yi-Shing Shieh, Huey-Kang Sytwu, Yuan-Wu Chen

**Affiliations:** ^1^ Graduate Institute of Life Sciences, National Defense Medical Center, Taipei, Taiwan; ^2^ Department of Oral and Maxillofacial Surgery, Tri-Service General Hospital, Taipei, Taiwan; ^3^ Division of Anatomic Pathology, Taipei Tzu Chi Hospital, Taipei, Taiwan; ^4^ Department of Biology and Anatomy, National Defense Medical Center, Taipei, Taiwan; ^5^ Department of Pharmacy Practice, Tri-Service General Hospital, Taipei, Taiwan; ^6^ School of Dentistry, National Defense Medical Center, Taipei, Taiwan; ^7^ Graduate Institute of Microbiology and Immunology, National Defense Medical Center, Taipei, Taiwan; ^8^ Department of Medical Research, Tri-Service General Hospital, Taipei, Taiwan

**Keywords:** patient-derived tumor xenograft, LSD1, melatonin, oral cancer, cell proliferation

## Abstract

Aberrant activation of histone lysine-specific demethylase (LSD1) increases tumorigenicity; hence, LSD1 is considered a therapeutic target for various human cancers. Although melatonin, an endogenously produced molecule, may defend against various cancers, the precise mechanism involved in its anti-oral cancer effect remains unclear. Patient-derived tumor xenograft (PDTX) models are preclinical models that can more accurately reflect human tumor biology compared with cell line xenograft models. Here, we evaluated the anticancer activity of melatonin by using LSD1-overexpressing oral cancer PDTX models. By assessing oral squamous cell carcinoma (OSCC) tissue arrays through immunohistochemistry, we examined whether aberrant LSD1 overexpression in OSCC is associated with poor prognosis. We also evaluated the action mechanism of melatonin against OSCC with lymphatic metastases by using the PDTX models. Our results indicated that melatonin, at pharmacological concentrations, significantly suppresses cell proliferation in a dose- and time-dependent manner. The observed suppression of proliferation was accompanied by the melatonin-mediated inhibition of LSD1 in oral cancer PDTXs and oral cancer cell lines. In conclusion, we determined that the beneficial effects of melatonin in reducing oral cancer cell proliferation are associated with reduced LSD1 expression *in vivo* and *in vitro*.

## INTRODUCTION

Oral cancer has a disfiguring, disabling, and painful prognosis [1, 2]. It can profoundly affect essential daily functions, such as food consumption, speech, sight, and hearing; these insults are compounded by distortions in facial appearance. Despite improvements in surgery and chemotherapy, the prognosis of advanced oral cancer remains poor.

Abnormal epigenetic changes including DNA methylation and histone modifications are hallmarks of cancer in humans [3]. Histone lysine-specific demethylase 1 (LSD1, also known as KDM1A) was the first identified FAD-dependent histone demethylase; it participates in gene regulation as a nuclear homolog of amine oxidases, specifically in demethylation of monomethylated and dimethylated histone 3 (H3) at lysine 4 (H3K4me 1/2) and of H3 at lysine 9 (H3K9me 1/2) [4, 5] and in demethylation of other nonhistone substrates, such as p53 and DNA (cytosine-5-)-methyltransferase 1 in some biological situations [6]. LSD1-overexpressing tongue squamous cell carcinoma is associated with tumor size, pathological grade, and reduced overall survival [7]. LSD1 has a major role in cancer development and progression and has therapeutic potential [8–10]; therefore, optimizing drugs for the treatment of this oral cancer type is crucial.

Melatonin (N-acetyl-5-methoxytryptamine), a natural compound present in animals, plants, and microbes [11], is effective against various cancers [12–14]; however, its effects against oral cancer are unclear. In addition, melatonin has anxiolytic, analgesic, and antidepressant effects, and it increases sleep efficiency [15, 16]. We suspected the clinical benefit may improve if melatonin is used for cancer therapy. Moreover, melatonin can induce H3 acetylation [17, 18]. In addition, inhibition of LSD1 activity or suppression of LSD1 expression enhances H3 acetylation [19]. Thus, melatonin may possess strong LSD1-associated anticancer characteristics.

A lack of appropriate preclinical models is mainly impeding the progress of oncological drug research. Patient-derived tumor xenograft (PDTX) models can offer several advantages compared with standard cell-line xenograft models in preclinical trials of novel anticancer drugs because PDTXs have the molecular, genetic, and histological heterogeneity typical of the tumors of origin propagated through serial passaging in mice [20, 21]. We recently established oral cancer PDTX models. Here, we examined the LSD1 status in oral cancer by using human tumor tissue arrays. We evaluated the anti-oral cancer effects of melatonin through suppression of LSD1 expression in our PDTX models *in vivo* and *in vitro*.

## RESULTS

### LSD1 is a potential novel diagnostic marker and therapeutic target in OSCC

**Figure 1:**
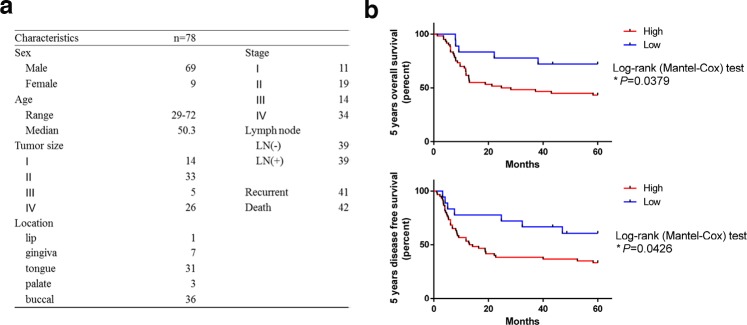
Overexpression of LSD1 is linked to poor outcome in oral cancer **(a)** Clinicopathological data of the 78 oral cancer patients used in the study. **(b)** The Kaplan-Meier curve compares the 5 years overall survival and 5 years disease free survival of cancer with high or low level LSD1 protein products. Definition of LSD1 high is samples that have IHC scores equate or above 4.

**Figure 2:**
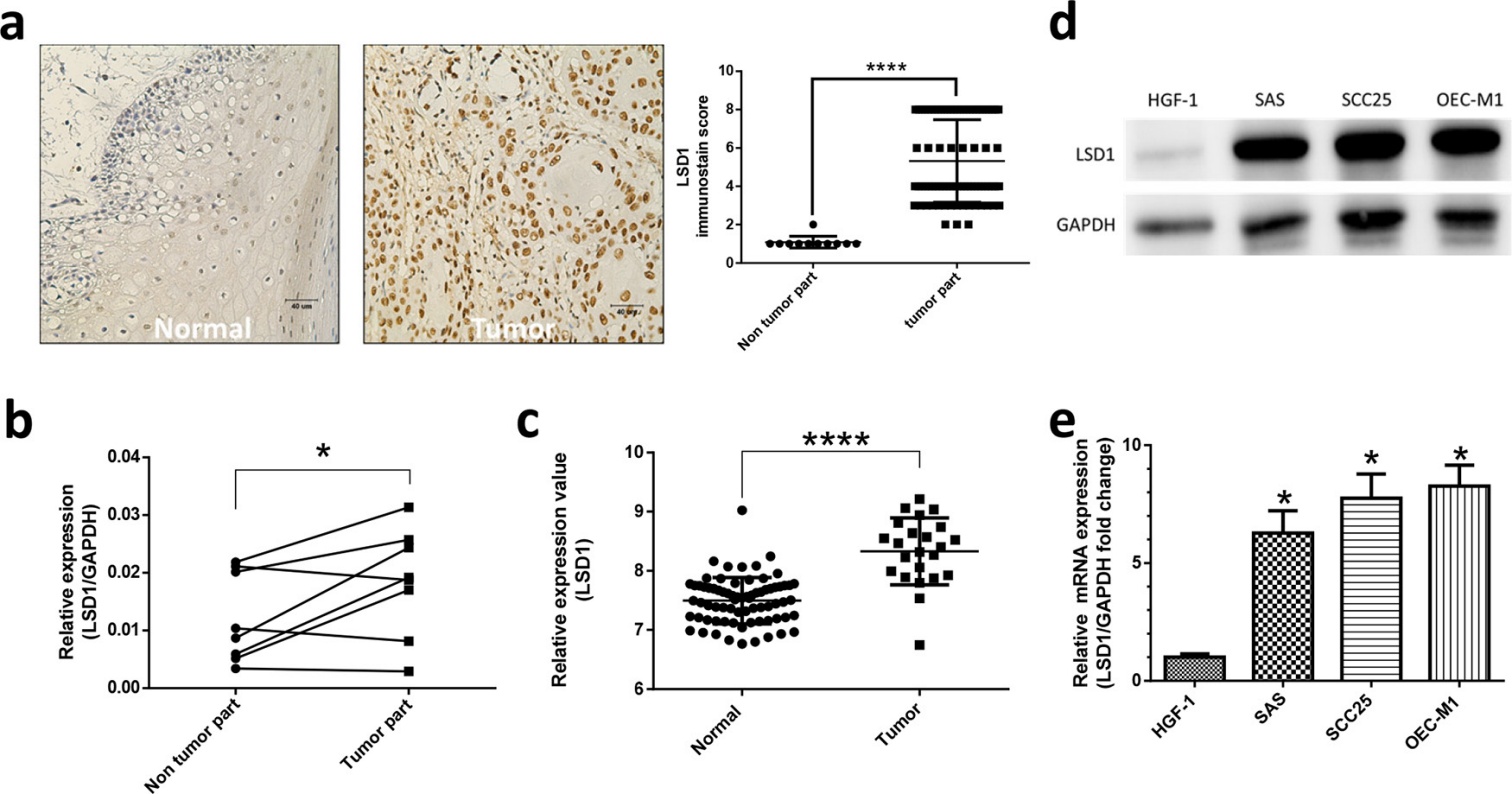
High expression of LSD1 existed in oral cancer **(a)** Positive nuclear immunostaining of LSD1 in normal mucosal and oral cancer tissues. **(b)** Q-PCR results from oral cancer tissues from eight patients and their eight matched normal mucosal tissues. **(c)** LSD1 mRNA expression in human tongue cancer. Data were obtained from NCBI Gene Expression Omnibus profiles (http://www.ncbi.nlm.nih.gov/geoprofiles; Reporter: GDS4562). LSD1 protein and mRNA levels in four oral cancer cell lines were determined through western blot analysis **(d)** and Q-PCR **(e)**, respectively. Normal human gingival fibroblast HGF-1 cells were used as the negative control. Data are presented as the mean ± SD. *P < 0.05 (Student *t* test).

LSD1 has an oncogenic role in various malignancies [22]. To assess the status of LSD1 in oral cancers, we evaluated the expression of LSD1 by using oral cancer tissue arrays (n = 78; Figure 1a, Supplementary Table 1, 2) containing different oral cancer grades as well as normal mucosal tissues and the percentage of stained (positive) cells was calculated as described previously [23]. LSD1 expression was significantly higher in oral cancer tissue than in normal oral mucosa (*P*< 0.0001; Figure 2a); it was positively correlated with survival rate and disease free survival rate (Figure 1b). We further analyzed LSD1 mRNA levels in OSCC tissues from eight patients and their eight matched normal mucosal tissues. Among these, five (62.5%) patient samples displayed higher LSD1 RNA levels in the OSCC tissues than in the adjacent normal mucosal tissues (*P* = 0.0434; Figure 2b). We also used a bioinformatics databank (NCBI Gene Expression Omnibus profiles) to assess the expression of LSD1 in tongue cancer and observed that LSD1 RNA levels were higher in OSCC tissues than in adjacent normal mucosal tissues (*P* < 0.0001; Figure 2c). Western blot and Q-PCR analyses of oral cancer cell lines displayed higher LSD1 expression in most of the tested oral cancer cell lines (Figure 2d, 2e). These results suggest that LSD1 is highly expressed in OSCC, demonstrating its potential as a novel diagnostic marker and therapeutic target.

### Histological and molecular characterization of oral cancer PDTX models

**Figure 3:**
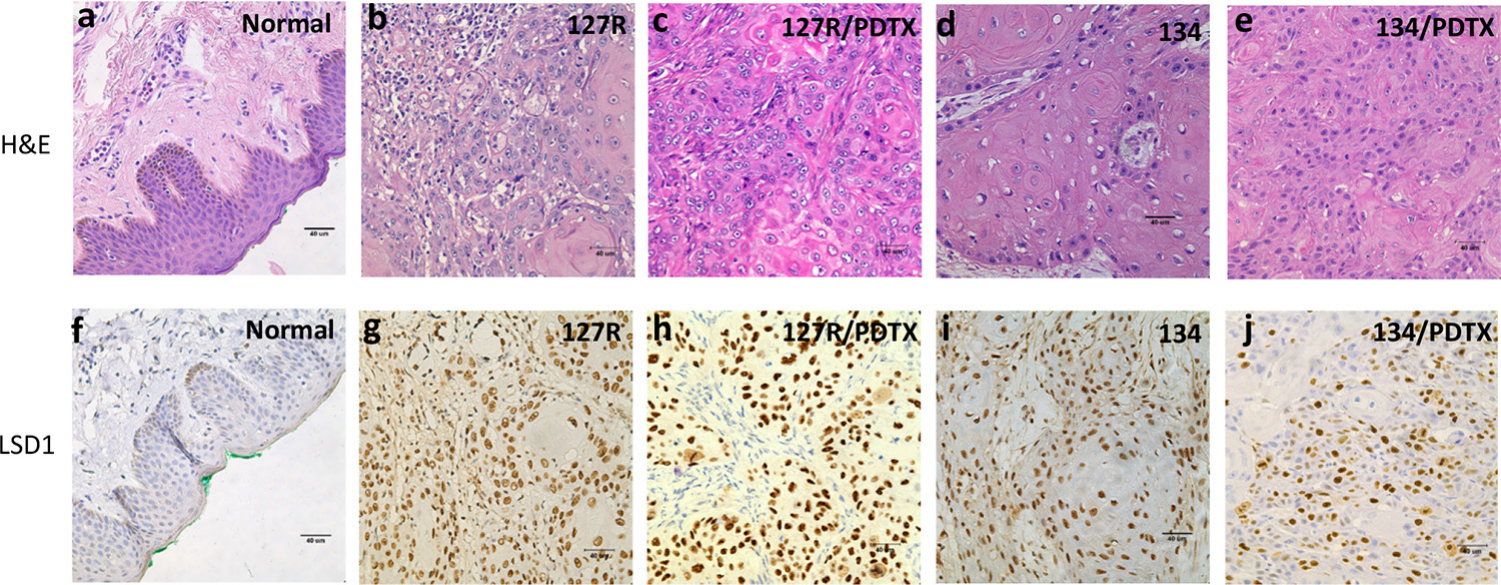
Histological and molecular characterization of the oral cancer PDTX models H&E staining of normal oral mucosa **(a)**, 127R clinical sample **(b)**, 127R PDTX model **(c)**, 134 clinical sample **(d)**, and 134 PDTX sample **(e)**. LSD1-specific immunohistochemical staining of normal oral sample **(f)**, 127R clinical sample **(g)**, 127R PDTX model **(h)**, 134 clinical sample **(i)**, and 134 PDTX sample **(j)**. Immunodetectable proteins are stained brown; nuclei are counterstained blue. Original magnification, 400×.

We successfully established oral cancer PDTX models; histological examinations employing H&E sections from the generation 4 xenografts of both 127R and 134 PDTX models (Figure 3c, 3e) revealed poorly differentiated OSCC, which was consistent with the original clinical cancer tissues (Figure 3b, 3d). Next, LSD1 expression was evaluated through immunohistochemical analysis: both clinical samples and PDTXs of 127R and 134 models displayed LSD1 overexpression (Figure 3g–3j).

### Melatonin inhibited tumor growth in oral cancer PDTX models

**Figure 4:**
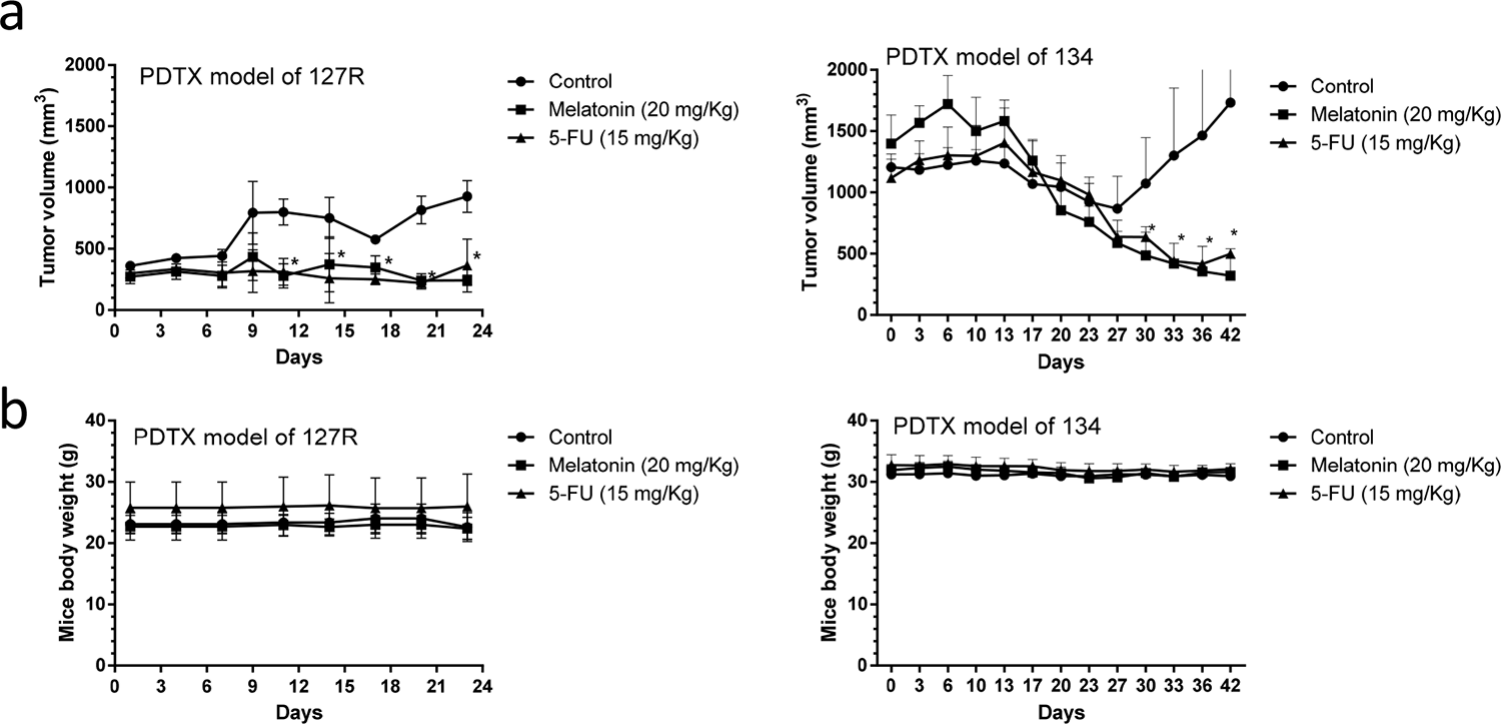
Melatonin inhibited tumor growth in LSD1-overexpressing oral cancer PDTX models **(a)** Changes in tumor volume in 127R and 134 oral cancer PDTX models, which were treated for 24 and 42 days, respectively, with melatonin (20 mg/kg daily i.p.), 5-FU (15 mg/kg), and PBS (a vehicle control). Diameters were measured twice a week for 24 or 42 days by using Vernier calipers, and the tumor volume was calculated as 1/2 × L × W^2^, where W and L are the shortest and longest diameters, respectively. Tumor volumes were compared with those of controls. All data are expressed as mean ± SD. *P < 0.05 (Student *t* test). **(b)** No significant change was observed in mice body weight compared with that of the vehicle control.

Melatonin is effective against various cancers, but its action mechanism against oral cancer is unclear. In this study, we further examined the effects of melatonin on growth in the oral cancer PDTX (preclinical) models. We observed that melatonin significantly inhibits tumor growth compared with the vehicle (PBS) control both in 127R and 134 oral cancer PDTX models (Figure 4a), where 5-FU was the positive control. No apparent toxicity or weight loss in the mice was observed after melatonin administration during the experimental period (Figure 4b).

### Melatonin repressed LSD1 expression in oral cancer PDTX models

**Figure 5:**
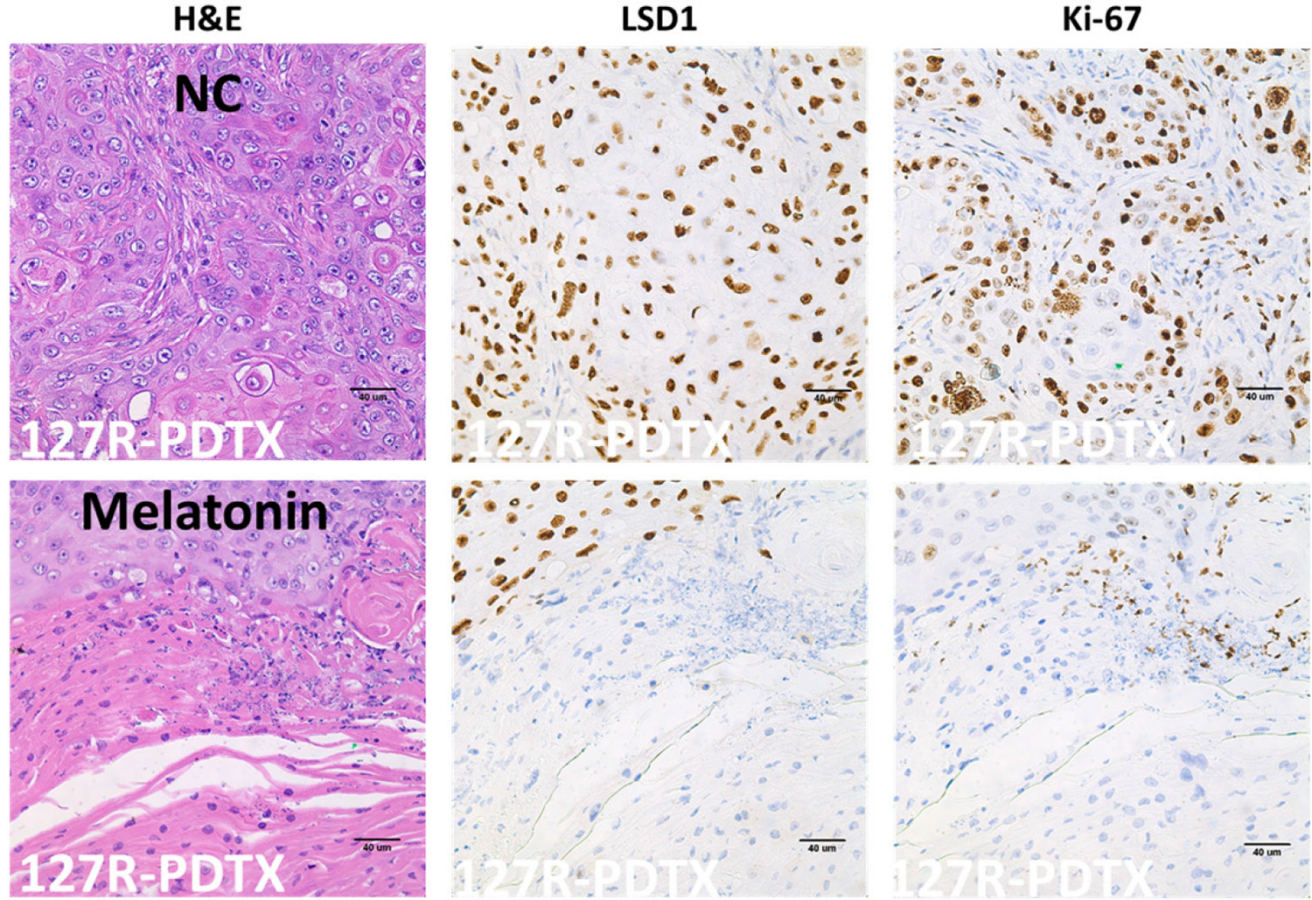

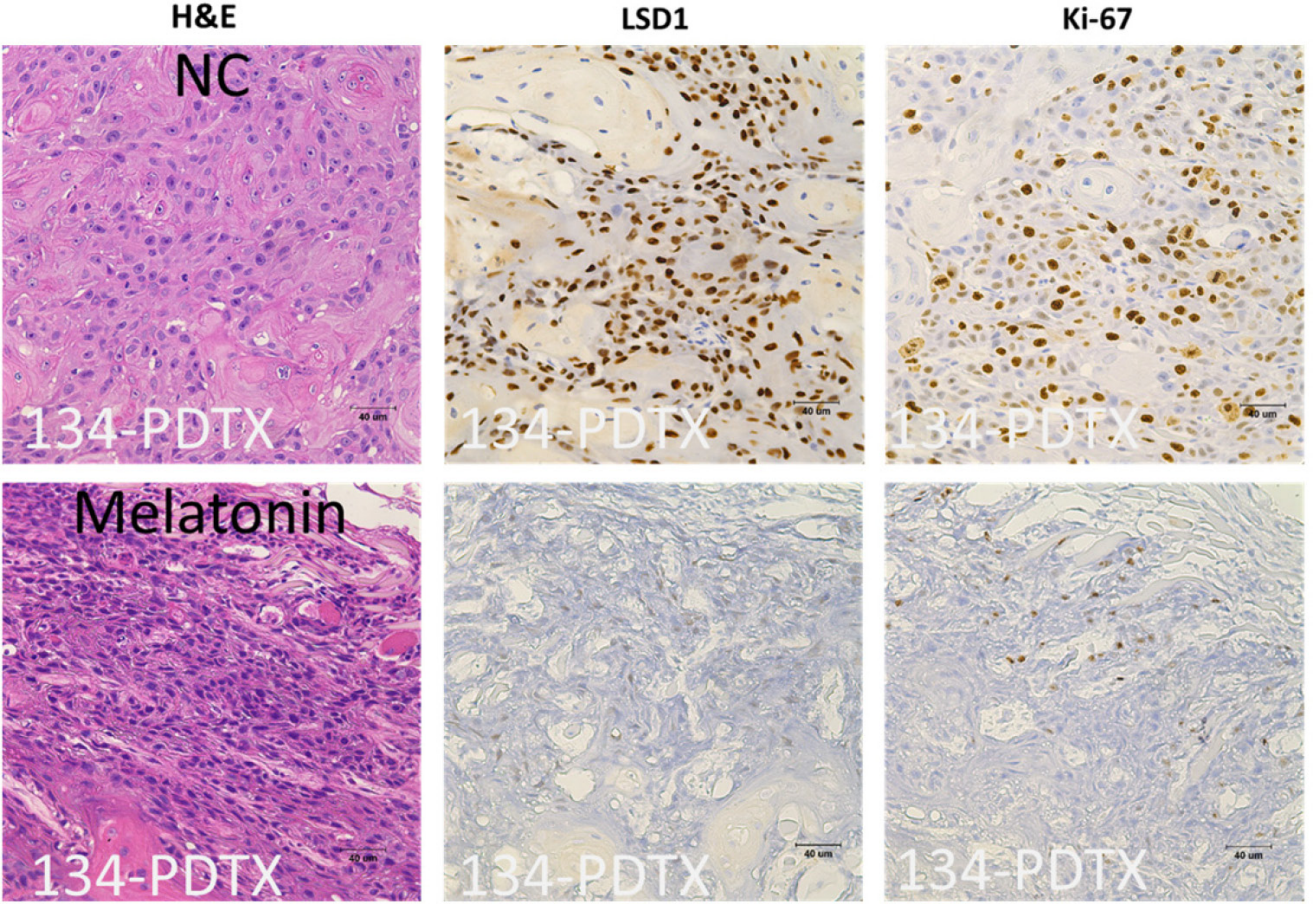
Effects of melatonin administration on the expressions of LSD1 and Ki-67 in oral cancer PDTX models H&E staining and IHC were performed after administration of melatonin or PBS (a vehicle control). Both 127R **(a)** and 134 **(b)** oral cancer PDTX models stained positive for LSD1 and Ki-67. Immunodetectable proteins are stained brown; nuclei are counterstained blue. Original magnification, 400×.

Abnormal activation of LSD1 signaling contributes to tumor survival, growth, and metastasis. In IHC analysis, we verified the LSD1 and Ki-67 expression in oral cancer PDTXs and observed that this expression decreases after melatonin administration in clinical tumor tissue-bearing mice compared with that in controls (Figure 5a, 5b). We observed that LSD1 expression significantly decreased in the melatonin-treated groups in both 127R and 134 PDTX models (Figure 5a, 5b). The expressions of Ki-67 (as proliferation maker) also highly decreased in the melatonin-treated group (Figure 5a, 5b).

### Melatonin decreased tumor growth and LSD1 expression in oral cancer xenograft model

**Figure 6:**
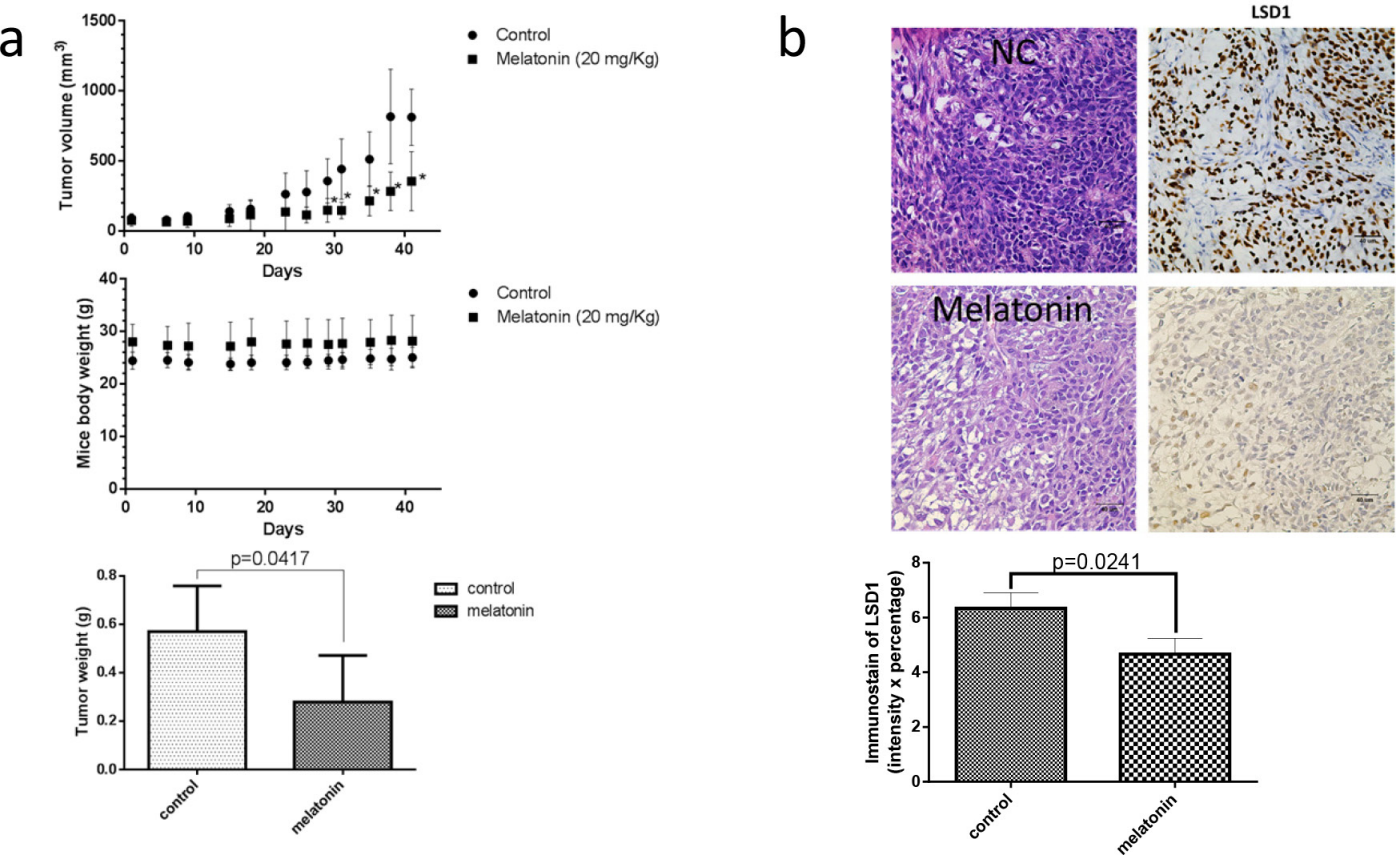
Melatonin inhibited oral cancer growth in SCC25 xenograft **(a)** SCC25 cells were subcutaneously xenografted into NOD/SCID mice (n = 5 per group). The tumor size was analyzed 42 days after administration of melatonin or PBS (a vehicle control). **(b)** H&E staining and IHC staining for LSD1 were performed after administration of melatonin or PBS (as a vehicle control). Immunodetectable proteins are stained brown; nuclei are counterstained blue. Original magnification, 400×. Data are represented as mean ± SD. *P < 0.05 (Student *t* test).

To verify the therapeutic effect of melatonin inhibition on the growth of oral cancer cells *in vivo*, we applied a mouse-based subcutaneous oral cancer xenograft model. NOD/SCID mice were subcutaneously implanted with 2 × 10^6^ SCC25 cells in their flank regions. Three days after injecting tumor cells, the mice were administered melatonin or PBS through i.p. injection. Tumor volumes were measured two times weekly, and mice were euthanized 42 days after treatment. Compared with PBS treatment, melatonin treatment significantly reduced the tumor growth and tumor weight (Figure 6a). No toxicities were observed in the treatment group, as determined through behavioral changes; mouse body weight did not differ significantly between the control and melatonin-treated groups (Figure 6a). LSD1 expression was significantly lower in the melatonin-treated group than in the controls (Figure 6b). These results suggested that melatonin-treated reduction in xenograft tumor growth involves reduced LSD1 expression and oral cancer cell growth.

### Melatonin and LSD1 inhibitor suppressed tumor growth in oral cancer cells

**Figure 7:**
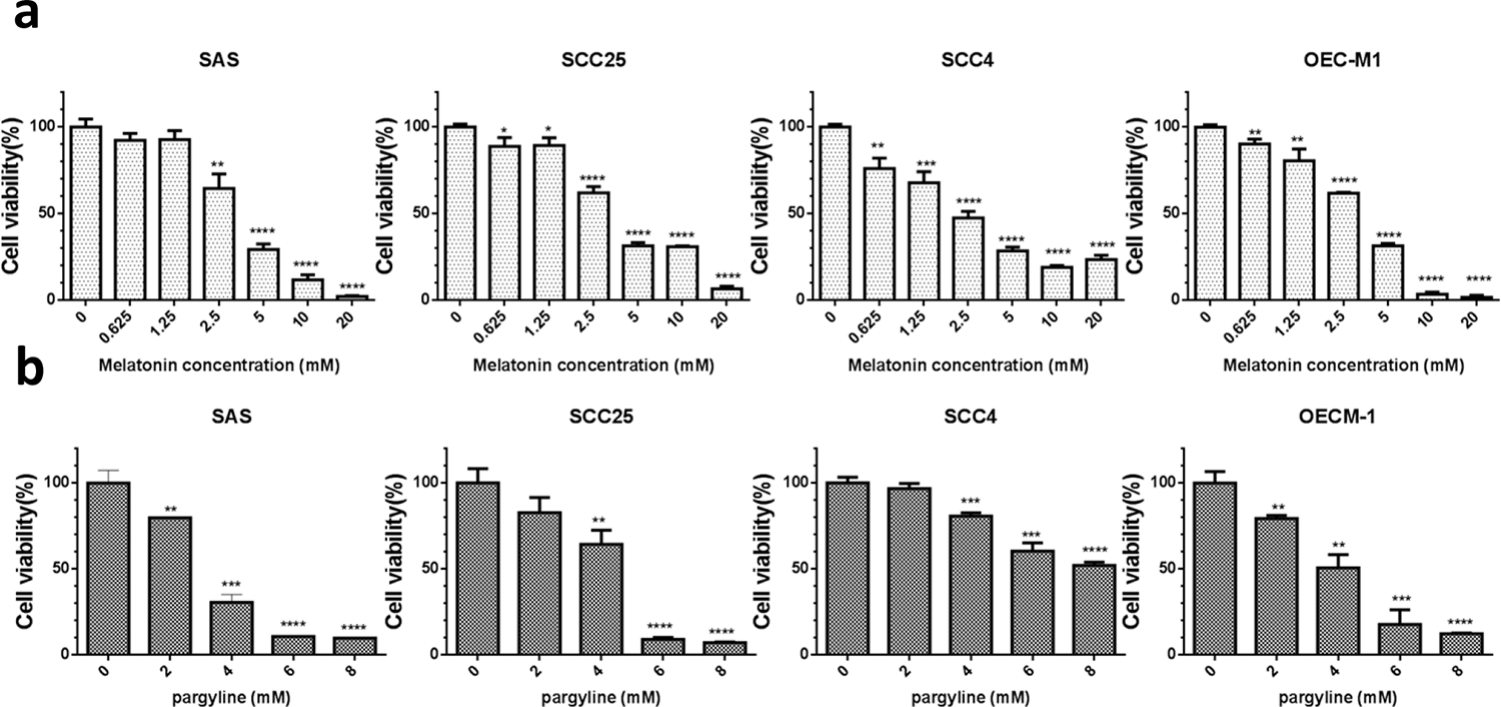
Melatonin inhibited oral cancer growth *in vitro* **(a)** Assessment of cell proliferation and viability by using the methylene blue assay in four oral cancer cells treated with varying concentrations of melatonin (0–20 mM) or DMSO (1 μL/mL) for 24 h. **(b)** Assessment of cell proliferation and viability by using the MTT assay in four oral cancer cells treated with varying concentrations of pargyline (0–8 mM) or DMSO (1 μL/mL) for 24 h. Data are represented as mean ± SD. *P < 0.05 (Student's *t* test).

To examine the significance of LSD1 in oral cancer cells, we used a pharmacological inhibitor of LSD1 activity, pargyline [24]. The treatment of oral cancer cells with melatonin or pargyline significantly reduced their proliferation in a dose-dependent manner (Figure 7). These results suggested that inhibition of LSD1 can potentially impair oral cancer cell proliferation.

### Melatonin induced oral cancer cells G0/G1 arrest

**Figure 8:**
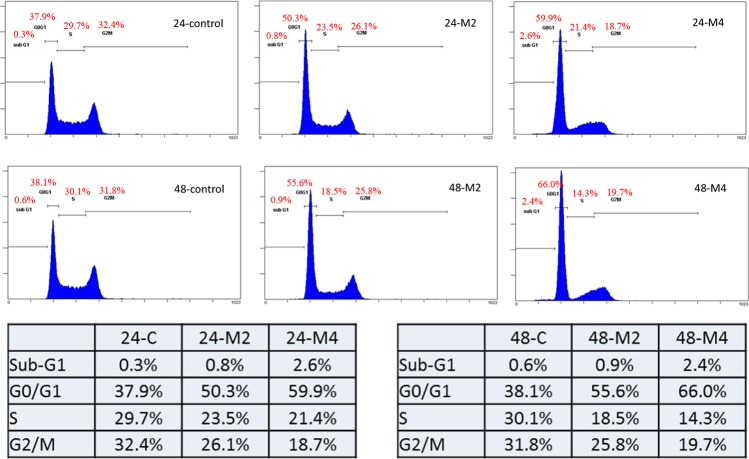
Melatonin induced oral cancer G0/G1 arrest Flow cytometric cell cycle analysis of SAS cells treated with varying concentrations of melatonin (2–4 mM) or DMSO (1 μL/mL) for 24 and 48h.

Melatonin repressed LSD1 expression and inhibited oral cancer cell growth *in vivo*. To explore the anticancer mechanism of melatonin, we analyzed the cell cycle through flow cytometry. Our results revealed that melatonin induces G0/G1 cell cycle arrest in oral cancer cells (Figure 8).

### Melatonin decreased LSD1 and cyclin D1 expression and increased p21 expression in oral cancer cells

**Figure 9:**
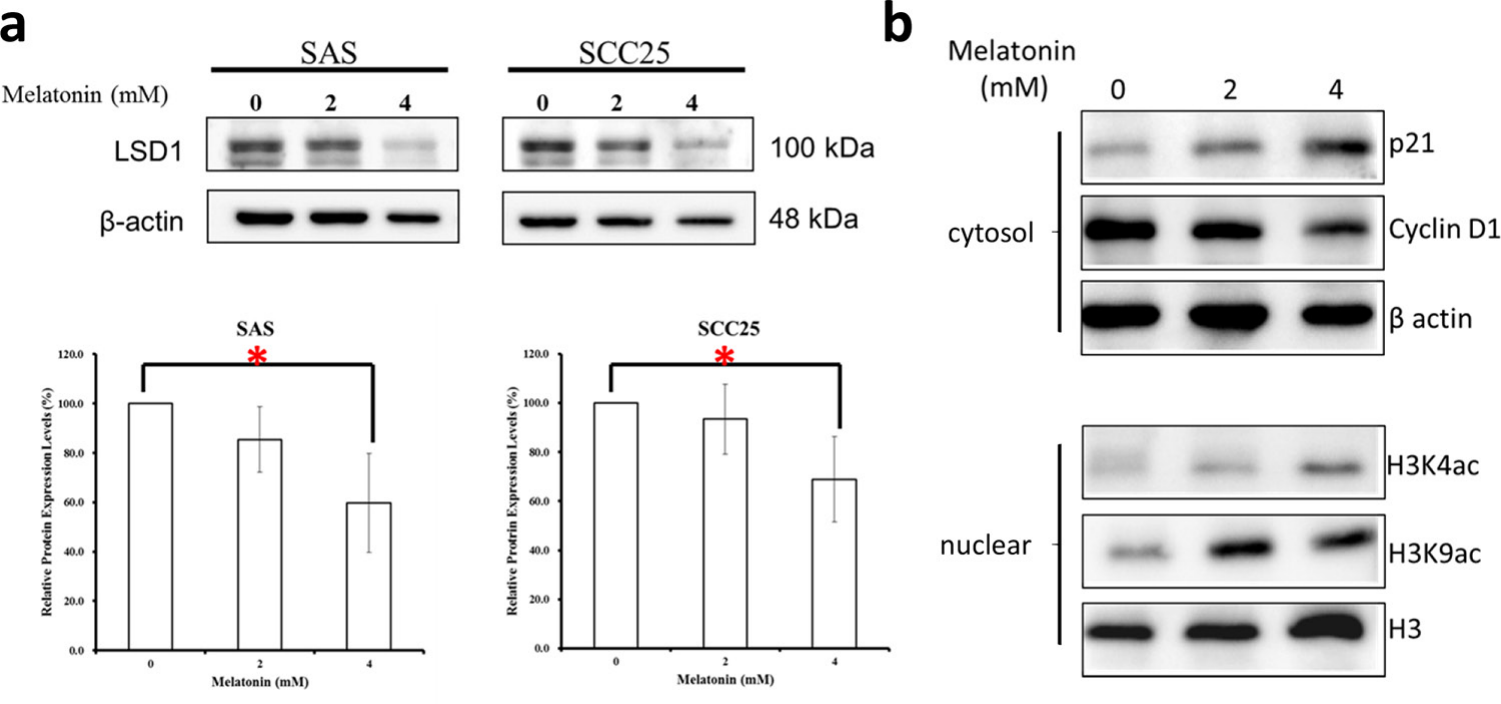
Melatonin repressed LSD1 expression in oral cancer cells **(a)** Western blot analysis and densitometry for LSD1 after SAS and SCC25 cells treated with melatonin for 24 h. **(b)** Western blot analysis of cyclin D1 and p21 expression after SCC25 cells treated with melatonin for 24 hrs. Data are presented as mean ± SD. *P < 0.05 (Student *t* test). The presented blots are representatives of those from at least three independent experiments.

Melatonin induces cell growth arrest by increasing p21 expression [25, 26]. Furthermore, LSD1 knockdown significantly increases p21 expression and induces cell cycle arrest [27]. Because LSD1 inhibition reduced oral cancer cell proliferation in our study, we examined whether melatonin reduces LSD1 expression in oral cancer cells. Melatonin substantially reduced LSD1 levels in both SAS and SCC25 cells (Figure 9a). Accordingly, western blot analysis revealed that treatment with melatonin significantly increased p21 and decreased cyclin D1 levels in SCC25 cells (Figure 9b). These studies suggested that blocking the LSD1 axis with melatonin can potentially reduce oral cancer cell proliferation and that melatonin likely exerts tumor-repressive effects by suppressing LSD1 expression.

### Melatonin induced H3K4/H3K9 acetylation

The current study revealed that melatonin induces H3 acetylation. To assess whether melatonin exerts an epigenetic role in oral cancer, we analyzed the H3 modification site in oral cancer. Our results revealed that melatonin induces H3 acetylation at H3K4 and H3K9 in oral cancer cells (Figure 9b).

## DISCUSSION

Melatonin is a marvelous versatility hormone which is synthesized and secreted by the pineal gland and shows both a wide distribution within organisms from bacteria to humans [28–31]. Studies have demonstrated that melatonin embraces anti-oral cancer effects, including regulating cancer cell evading apoptosis, angiogenesis, metastasis, evading the immune system, and inflammation [28, 32–37]. Recent study demonstrated that melatonin can protect against radiation-induced oral mucositis (Supplementary Figure 4), which is a particularly severe and common acute side effect of chemotherapy or radiotherapy [34]. A surprising benefit of melatonin treatment is its few side effects; it relieved depressive symptoms, anxiety, insomnia, reduced cognitive function, and fatigue as observed in cancer patients being treated with other related therapies [15]. In this study, we evaluated the response of human oral cancer to melatonin treatment in patient-derived tumor xenograft, *in vivo* and *in vitro*. Melatonin effectively inhibited cell proliferation. Our results indicated that melatonin suppressed LSD1 expression (Figure 9a), reduced its mRNA levels, and induced H3K4 and H3K9 acetylation (Figure 9b). Melatonin also induced cell cycle arrest in the G0/G1 phase (Figure 8, Figure 9b). Melatonin can inhibit cancer cell growth by modulating epigenetic effects through DNA methylation and histone acetylation pathways [12, 17, 18, 38, 39]. Melatonin also induces H3 hyperacetylation in neural stem cells [18]. According to our results, melatonin can induce histone acetylation at H3K4 and H3K9 (Figure 9b). Moreover, H3K4 or H3K9 acetylation is typically associated with gene transcription [40, 41]. These findings indicate that the antioral cancer effect of melatonin is attributable to the alteration of the H3K4 or H3K9 acetylation.

In past decades, aberrant epigenetic dysregulation was considered the key indication of human cancer [3, 42]. Epigenetic alterations are generally reversible and dynamically regulated; thus, they may be novel therapeutic targets for cancer therapy [43, 44]. The increasing evidence has linked diverse histone modifications to cancer initiation and progression [45]. Moreover, H3K4 acetylation levels may facilitate diagnosis or therapy of oral cancer: low H3K4 acetylation levels in oral cancer indicate advanced tumor stage and poor prognosis [46]. LSD1, a H3K4 or H3K9 demethylase, regulates gene expression pathways by altering the epigenetic histone markers in gene promoters [47]. Epigenetic alterations in LSD1 have a major role in tumorigenicity [48, 49] and are associated with poor prognosis [7, 8, 50–55]. Inhibition of LSD1 activity or repression of LSD1 expression can inhibit tumor cell growth (Supplementary Figure 3) and induce histone acetylation [19]. High LSD1 expression levels in tongue cancer are associated with proliferation and poor prognosis [7]. Here, we verified LSD1 expression by using a clinical oral cancer tissue microarray (n = 78) and oral cancer cell lines (n = 4); we also assessed its clinicopathological significance in oral cancer patients. Our results indicated that LSD1 is aberrantly overexpressed in oral cancer, and this overexpression is associated with poor prognosis (Figure 1). The aggressive nature of LSD1-overexpressing oral cancer and lack of effective therapeutic options make clinical oral cancer treatment particularly challenging; thus, developing novel therapies is crucial. However, research in this area has been impeded by lack of clinically relevant experimental models.

Here, we determined the antitumor effects of melatonin in LSD1-overexpressing oral cancer PDTXs and oral cancer cell line xenograft models. To our knowledge, this is the first study of melatonin in PDTX models. Our results indicated that melatonin exerted a substantial antitumor effect on PDTX and traditional xenograft models of oral cancer (Figure 4). The PDTX mouse model accurately represents human tumors through increased diversity of molecular lesions and preservation of three-dimensional tumor-stromal cell components and interactions [56]. Hence, these models have been increasingly used as tools for the preclinical assessment of anticancer drugs. Some PDTX models (e.g., lung cancer [57–59], melanoma [60], colon cancer [61], breast cancer [62], hepatocellular carcinoma [63, 64], and gastrointestinal stromal tumor [65] have been established and used for evaluating antitumor compounds [66]; however, the availability of oral cancer PDTX models is limited. In this study, we used the NSG mice to establish oral cancer PDTX models. The tumor growth rate differed among our PDTX models. Both 127R and 134 PDTX models were obtained from clinical advanced oral cancer patients with lymphoid metastasis. We performed the histological and molecular characterization of the tissues from oral cancer patients and PDTX tissues through immunochemistry of cells from generations 4 and 7 of the 127R and 134 PDTX models, respectively. Our results showed that LSD1 is overexpressed in oral cancer tissues of both clinical patients and PDTX (Figure 3). As per the H&E staining results, histology of the PDTX models was similar among all cancer patient samples. We will establish more PDTX lines to further clarify the abundant tumor heterogeneity in future studies.

Our results revealed that melatonin has anticancer effects similar to 5-FU (Figure 4). Anticancer drugs cause severe side effects, including cardiotoxicity. For example, 5-FU and cisplatin are major chemotherapeutic agents in the treatment of head and neck cancer. Despite its proven therapeutic efficacy, 5-FU and cisplatin (Supplementary Figure 1) also possess several undesirable cardiotoxicities, including coronary vasospasm, coronary thrombosis, cardiomyopathy, and sudden cardiac death [67]. In contrast to many anticancer drugs on the cardiomyocytes, melatonin has low toxicity [68] and has beneficial effects against cardiotoxicity [69]. Based on previous studies, we compared the anticancer effects of 5-FU and melatonin on oral cancer PDTX models.

Our data suggested that melatonin is a potential therapeutic option for LSD1-overexpressing oral cancer, but the detailed action mechanism of melatonin in oral cancer requires further detailed investigation. Our *in vitro* study revealed that melatonin or an LSD1 inhibitor, pargyline, strongly inhibit the proliferation of tongue (SAS, SCC25, and SCC4) and gingival (OEC-M1) cancer cells. Our *in vivo* IHC assay demonstrated that melatonin strongly reduces LSD1 and Ki-67 expression (Figure 5a, 5b); consistent with this, in our *in vitro* study, melatonin significantly downregulated LSD1 (Figure 9a) and induced H3K4 methylation (Supplementary Figure 2). These data suggest that melatonin is a potential therapeutic option for LSD1-overexpressing oral cancer, but the detailed action mechanism of melatonin in oral cancer requires further detailed investigation.

In conclusion, we demonstrated that the anti-oral cancer effect of melatonin is accompanied by LSD1 downregulation *in vitro*, *in vivo*, and in our preclinical PDTX model. Thus, LSD1 can be a diagnostic marker and therapeutic target for oral cancer. Furthermore, melatonin can potentially be an effective chemotherapeutic agent for oral cancer. Finally, this study lays a foundation for further evaluation of the action mechanism of melatonin against oral cancer.

## MATERIALS AND METHODS

### Tissue microarray

Microarray slides of OSCC tissue were prepared using 78 paraffin-embedded primary OSCC tumors and 11 normal oral mucosal tissues (Figure 1A). Two core tissue samples were extracted from a representative area of each paraffin-embedded tumor tissue. Each representative core sample in the tissue microarray slide measured 2 mm in diameter. This microarray study was approved by the ethics review committees of Tri-Service General Hospital, Taiwan (IRB: TSGH-2-102-05-111 and TSGH-2-105-05-002). All patients were not received surgery, radiotherapy, or chemotherapy previously.

### Histology and immunohistochemistry

Mice were sacrificed using CO_2_, and their tissues were fixed through perfusion by using 4% paraformaldehyde in 0.1 M phosphate buffer. Next, 5-μm-thick serial histological sections were taken on slides, deparaffinized in xylene, and rehydrated. After blocking endogenous peroxidase by using 3% hydrogen peroxide, the slides were incubated with the anti-LSD1 and anti-Ki-67 antibodies overnight at 4°C. Target protein expression was detected using an anti-mouse and anti-rabbit peroxidase complex, and peroxidase activity was observed using 3-amino-9-ethyl-carbazole. The slides were counterstained with hematoxylin (Sigma–Aldrich) and mounted using a mounting solution. The immunohistochemistry (IHC) calculations are described in the next section.

### Evaluation of immunohistochemical staining

The intensity of tumor cell immunoreactivity was scored on a scale of 0–3: 0, no staining; 1, weak intensity; 2, moderate intensity; and 3, strong intensity. The percentage of tumor cells with nucleus staining for each intensity score was graded on a 5-point scale: 0, 0%; 1, 0- 25%; 2, 25%- 50%; 3, 50%- 75%; and 4, 75- 100% stained tumor cells. The immunostaining scores (ranged 0–12) were determined by multiplying scores based on the percentages of stained tumor cells (0–4) with the intensity scores (0–3). Definition of LSD1 high is samples that have IHC scores equate or above 4.

### Cell culture and reagents

Human tongue squamous cell carcinoma cell-line SAS was provided by Dr. Jeng–Fan Lo [70]. SCC4 (CRL-1624; ATCC) and SCC25 (CRL-1628; ATCC) were obtained from the American Type Culture Collection. The OEC-M1 was established using primary tumors obtained from adult male OSCC patients from Taiwan with a history of betel quid chewing. SAS, SCC25, and OEC-M1 cells were cultured in RPMI 1640, whereas SCC4 cells were cultured in DMEM/F12. All cultured media were supplemented with 10% fetal bovine serum, 1% penicillin/streptomycin, and 2 mmol/L L-glutamine, and all cells were grown at 37°C in a humidified incubator with 5% CO_2_ atmosphere.

Melatonin (M5250, purity of ≥ 98% determined through high performance liquid chromatography; Sigma–Aldrich) was dissolved in ethanol (95%) to form a 0.1 g/mL stock and added to cells at indicated concentrations. 5-FU (F6627; purity of ≥ 99% determined through high performance liquid chromatography; Sigma-Aldrich) was dissolved in saline to form a 1.5 mg/mL stock as the positive control in the animal models.

### Establishment of PDTX models and treatment protocol

Tumor specimens were obtained from oral squamous cell carcinoma (OSCC) patients with lymphatic metastases during initial surgery. The experiments were conducted according to the ethical guidelines of the institutional review board (TSGH-2-102-05-111) of the National Defense Medical Center, Taiwan. The patients had not received chemotherapy or radiotherapy before surgery. The histological type of all tumor specimens was T4aN2b, as per the World Health Organization criteria. The tumor samples were placed in RPMI 1640 under sterile conditions immediately after surgical resection and transported to the animal tissue culture facility.

The tumors were implanted subcutaneously into NOD/SCID/IL2R gamma null (or NOD.Cg-Prkdcscid Il2rgtm1Wjl/SzJ; NSG) mice. The NSG mice (aged 8–10 weeks) were obtained from Jackson Laboratory (USA) and maintained in the National Defense Medical Center, Taiwan. All experiments were approved by the Institutional Animal Care and Use Committee (IACUC) of the National Defense Medical Center (IACUC 14–299 and 15–028). Xenograft growth was monitored at least two times weekly: lengths (longest diameters) and widths (shortest diameters) of the tumors were measured using Vernier calipers, and the tumor volume was calculated using the following formula: volume = 1/2 × length × width^2^. When a tumor volume was approximately 3000 mm^3^, the tumor was removed for serial transplantation.

When the tumor volume reached approximately 500 mm^3^, mice with fourth (127R)- or seventh (134)-generation PDTXs were randomized into three groups (127R PDTX line, n = 3 per group; 134 PDTX line, n = 5 per group) to receive melatonin (20 mg/kg/daily), vehicle control (phosphate-buffered saline, PBS), and 5-fluorouracil (5-FU; 15 mg/kg/daily; positive control) through intraperitoneal (i.p.) injection in late afternoon for 24 (127R model) and 42 (134 model) days. Mouse weight and tumor volume were measured at least two times weekly. The transplanted tumor size was measured using Vernier calipers two times weekly, and the tumor volume was calculated using the aforementioned formula. At the end of the treatment, the mice were sacrificed, and the tumors were removed, weighed, and visualized.

### Xenograft tumor model

Six-week-old NOD.CB17 Prkdcscid/J mice (National Laboratory Animal Center, Taiwan) mice were maintained in a microisolator under pathogen-free conditions. Each mouse was subcutaneously injected with 2 × 10^6^ SCC25 cells, and the mice were then divided into two groups (n = 5 per group). After 3 days, one group was treated with melatonin (20 mg/kg daily i.p.) in late afternoon, whereas the other was treated with a vehicle (PBS i.p.). The size of the transplanted tumors was measured using Vernier calipers two times weekly, and the tumor volume was calculated using the aforementioned formula. At the end of the treatment, the mice were sacrificed, and the tumors were removed, weighed, and visualized.

### Cell growth inhibition assay

Cells (10,000/well in 24-well plates) were exposed to various concentrations of melatonin for 24–72 h. A methylene blue assay was used to evaluate the effect of melatonin on cell growth, as described previously [71]. The IC_50_ value, indicating the concentration of melatonin causing a 50% inhibition of cell growth, was calculated statistically by comparing it with that of controls.

### Quantitative real-time PCR

Total RNA was extracted from oral cancer cells by using the TRIzol Reagent (Invitrogen) according to the manufacturer's protocol. First-strand cDNA synthesis and amplification was performed using the Maxima H Minus First Strand cDNA Synthesis Kit (Thermo Scientific, Rockford, USA). The following quantitative real time PCR (Q-PCR) primers were designed using Primer3 (NCBI): LSD1, 5′-GCTCGGGGCTCTTATTCCTA-3′ (forward) and 5′-CCCAAAAACTGGTCTGCAAT-3′ (reverse); GAPDH, 5′-GGAAGGTGAAGGTCGGAGTCA-3′ (forward) and 5′-GTCATTGATGGCAACAATATCCACT-3′ (reverse). Q-PCR amplifications were performed on a real-time PCR system (Applied Biosystems 7500 Fast) by using 20-μL reaction volumes containing 15 μL of SYBR Green PCR Master Mix (Thermo Scientific). The changes in LSD1 expression were calculated using 7500 software (Version 2.0.6, Applied Biosystems).

### Cell cycle analysis

The cell cycle was assayed by propidium iodide (PI) staining, followed by Cytomics FC500 Flow Cytometer CXP analysis. Cell cycle profiles were then determined using the CXP analysis software (Beckman Coulter Inc., Miami, FL, USA).

### Western blot analysis

Cells were lysed directly in RIPA buffer containing 50 mM Tris (pH 7.8), 0.15 M NaCl, 5 mM EDTA, 0.5% Triton X-100, 0.5% NP-40, 0.1% sodium deoxycholate, a protease inhibitor mixture, and a phosphatase inhibitor mixture (Calbiochem, Billerica, MA, USA). The relative protein concentration in the supernatants was determined using a BCA protein assay kit (Thermo Scientific). For each lane of 10% SDS-PAGE, 30 μg of cell lysate protein was loaded, separated, and transferred onto a polyvinyldifluoride membrane (GE Healthcare, UK). The membranes were then probed using specific antibodies against LSD1 (A300-215A, Bethly), H3K4ac (07-539, Millipore), H3K9ac (ab4441, Abcam), H3K4me2 (ab32356, Abcam), H3 (H0164, Sigma-Aldrich), cyclin D1 (#1085-1, Epitomics), p21 (ab81283, Abcam) and β-actin (#3053-1, Epitomics).

### Statistical analysis

Data (mean ± SD, 2–3 experiments) were analyzed for statistical significance by using unpaired, two-tailed Student *t* tests. *P* < 0.05 was considered statistically significant. We performed statistical analyses by using GraphPad Prism (GraphPad Software, San Diego, CA, USA). All cell-line experiments were repeated a minimum of three times, unless stated otherwise. Survival data were analyzed with the Kaplan-Meier method, tested with the log-rank method and further analyzed for multiple factors using the Cox regression model. P<0.05 was considered to indicate a statistically significant difference.

## SUPPLEMENTARY MATERIALS FIGURES AND TABLES


