# Supplementary material for: Melatonin exerts anti-oral cancer effect via suppressing LSD1 in patient-derived tumor xenograft models
Source: Oncotarget. 2017 Apr 4;8(20):33756–69. doi: 10.18632/oncotarget.16808 (PMC5464909; doi:10.18632/oncotarget.16808)
Supplement: Supplementary file 1 [file oncotarget-08-33756-s001.pdf]

# Melatonin exerts anti-oral cancer effect via suppressing LSD1 in patient-derived tumor xenograft models

## SUPPLEMENTARY FIGURES AND TABLES

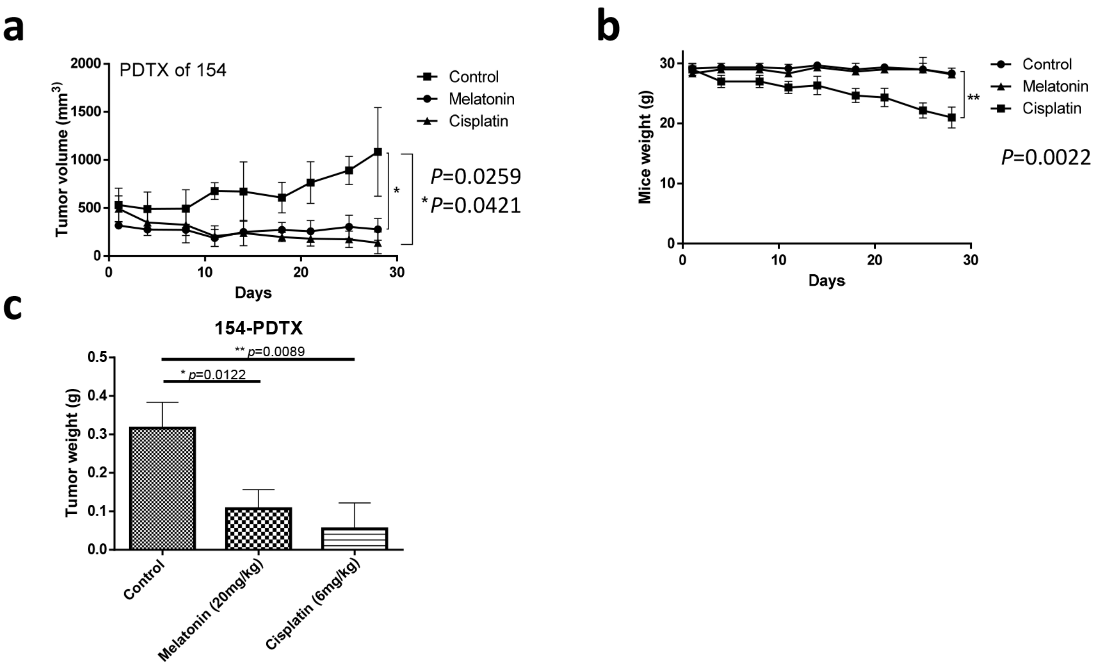


**Supplementary Figure 1: Cisplatin as the positive control *in vivo* study. (a)** Changes in tumor volume in 154 oral cancer PDTX model, which were treated for 28 days with melatonin (20 mg/kg daily i.p.), cisplatin (6 mg/kg), and PBS (a vehicle control). Diameters were measured twice a week for 28 days by using Vernier calipers, and the tumor volume was calculated as 1/2 × L × W2, where W and L are the shortest and longest diameters, respectively. Tumor volumes were compared with those of controls. All data are expressed as mean ± SD. *P < 0.05 (Student t test). **(b)** A significant change in cisplatin group was observed in mice body weight compared with that of the vehicle control. **(c)** Average tumor weight of each group was compared to controls. (*P<0.05 by Student's *t* test).

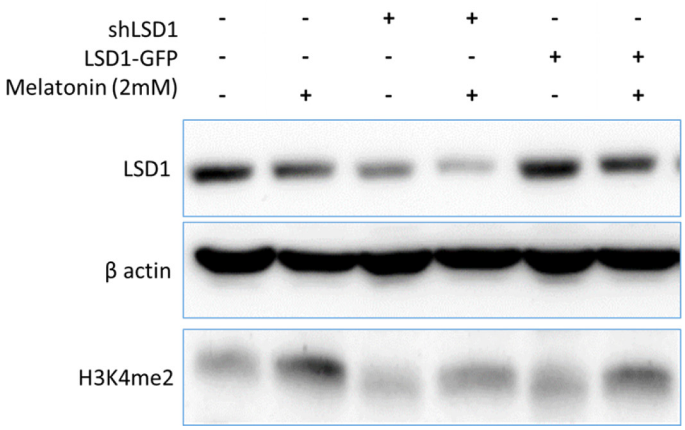


**Supplementary Figure 2: Melatonin repressed expression of LSD1 and induced expression of H3K4me2 in oral cancer cells.** Melatonin repressed expression of LSD1 and induced expression of H3K4me2. Western blot analysis for expression of LSD1 and H3K4me2 after melatonin treated with LSD1- overexpression or knockdown SAS cells for 24 h.

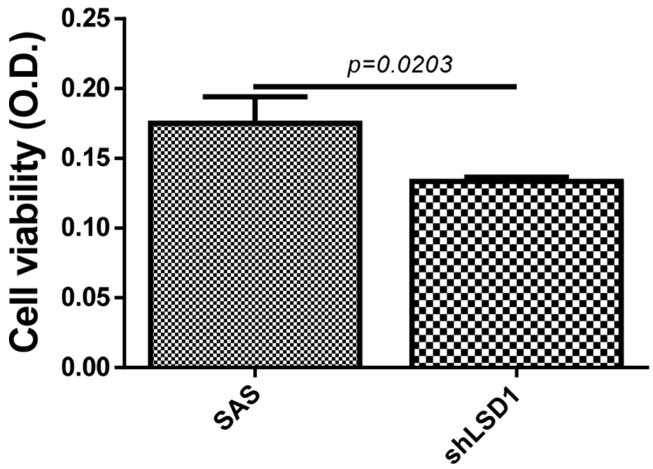


**Supplementary Figure 3: Repressed expression of LSD1 by shRNA was inhibited oral cancer growth.** Assessment of cell proliferation and viability by using the methylene blue assay in oral cancer cells SAS knockdown by shRNA of LSD1 for 24 h.

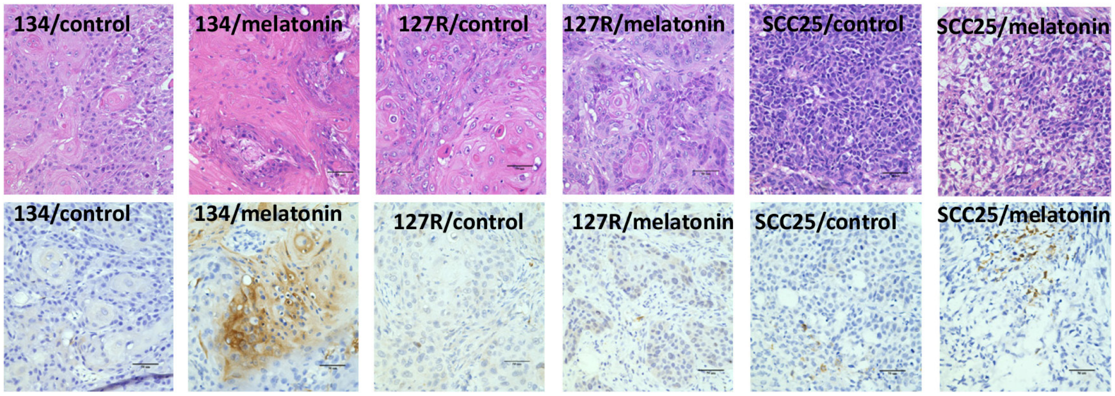


**Supplementary Figure 4: Melatonin induced expression of heme oxygenase-1 (HO-1) in PDTX and in oral cancer cells SCC25 xenografts model.** H&E staining and IHC were performed after administration of melatonin or PBS (a vehicle control). 127R, 134 oral cancer PDTX models and SCC25 xenografts model stained HO-1. Immunodetectable proteins are stained brown; nuclei are counterstained blue. Original magnification, 400×.

**Supplementary Table 1: LSD1 expression pattern in OSCC and normal oral mucosa**

| | LSD1 expression | | *p*-values |
|---|---|---|---|
| | **Low** | **High** | |
| Normal oral mucosa | 11 | 0 | **< 0.0001** |
| OSCC | 18 | 60 | |

**Supplementary Table 2: Associated between LSD1 expression and mutiple clinicopathological parameters in primary OSCC**

| Clinicopathological parameters | | Cases | LSD1 | | *p*-values |
|---|---|---|---|---|---|
| | | | Low | High | |
| Gender | | 78 | | | |
| | Male | 69 | 15 | 54 | 0.4234 |
| | Female | 9 | 3 | 6 | |
| Age | | | | | |
| | ≦50 | 42 | 12 | 30 | 0.2838 |
| | >50 | 36 | 6 | 30 | |
| Tumor size | | | | | |
| | T1-T2 | 47 | 11 | 36 | 1.0000 |
| | T3-T4 | 31 | 7 | 24 | |
| Cervical node metastasis | | | | | |
| | N(-) | 39 | 10 | 29 | 0.7887 |
| | N(+) | 39 | 8 | 31 | |
| Pathological grade | | | | | |
| | I | 19 | 3 | 16 | 0.5361 |
| | II-III | 59 | 15 | 44 | |
| Clinical stage | | | | | |
| | I-II | 30 | 6 | 24 | 0.7836 |
| | III-IV | 48 | 12 | 36 | |
